# Supplementary material for: Chemical space docking enables large-scale structure-based virtual screening to discover ROCK1 kinase inhibitors
Source: Nat Commun. 2022 Oct 28;13:6447. doi: 10.1038/s41467-022-33981-8 (PMC9616902; doi:10.1038/s41467-022-33981-8)
Supplement: Supplementary file 1 — Supplementary information [file 41467_2022_33981_MOESM1_ESM.pdf]

Supplementary Table 1: ROCK1 Actives

Pyrazole

Cpd #	Structure	ROCK1 K_i (μM)*
1	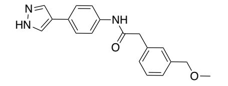	0.038 +/- 0.001
2	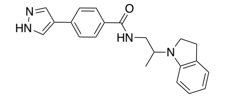	0.049 +/- 0.002
3	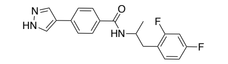	0.076 +/- 0.026
4	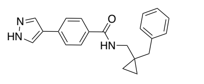	0.079 +/- 0.013
5	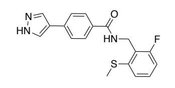	0.082 +/- 0.001
6	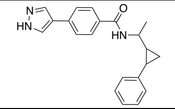	0.087 +/- 0.028
7	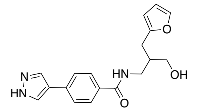	0.132 +/- 0.038
8	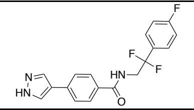	0.155 +/- 0.006
9	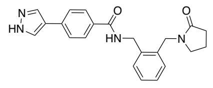	0.285 +/- 0.028
10	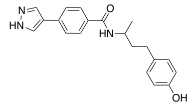	0.310 +/- 0.012
11	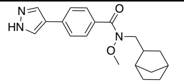	0.573 +/- 0.144
12	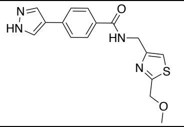	1.100 +/- 0.178
13	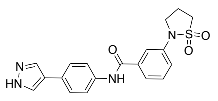	2.142 +/- 0.143
14	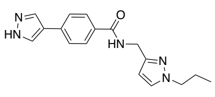	3.606 +/- 0.125
15	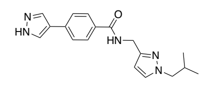	6.805 +/- 0.146

*Average +/- standard deviation of three measurements. Source data are provided as a Source Data file.

Lactam/Pyridone

Cpd #	Structure	ROCK1 K_i (μM)
16	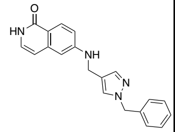	0.793 +/- 0.078
17	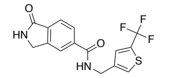	1.356 +/- 0.071
18	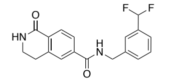	4.503 +/- 0.143
19	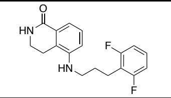	5.094 +/- 0.145
20	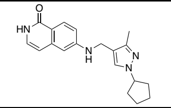	5.603 +/- 0.188
21	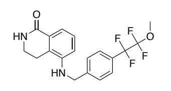	7.232 +/- 0.070

Azaindole

Cpd #	Structure	ROCK1 K_i (μM)
22	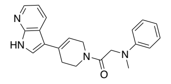	0.097 +/- 0.004
23	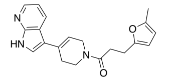	2.202 +/- 0.030
24	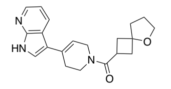	4.393 +/- 0.131

Indazole

Cpd #	Structure	ROCK1 K_i (μM)
25	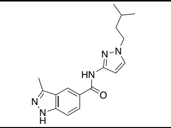	1.826 +/- 0.066
26	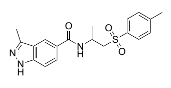	6.639 +/- 0.512
27	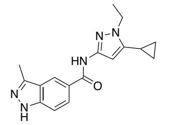	6.709 +/- 1.592

Supplementary Table 2-A: Chemical Characterization of ROCK1 Actives

Compound #	Enamine ID	1H NMR	13C NMR	HRMS (m/z)	Purity (HPLC area % at 254 nm)
1	Z4540472702	1H NMR (500 MHz, DMSO-d6) δ 12.86 (s, 1H), 10.13 (s, 1H), 8.09 (s, 1H), 7.92 (s, 0H), 7.86 (s, 1H), 7.62 – 7.48 (m, 4H), 7.34 – 7.28 (m, 2H), 7.28 – 7.22 (m, 1H), 7.19 (dt, J = 7.0, 1.6 Hz, 1H), 4.40 (s, 2H), 3.63 (s, 2H), 3.30 (s, 3H)	13C NMR (126 MHz, DMSO) δ 168.77, 165.17, 138.27, 137.06, 135.96, 135.87, 134.95, 130.43, 128.28, 128.17, 128.11, 127.95, 125.61, 125.28, 121.33, 120.82, 120.66, 120.15, 119.39, 73.51, 73.20, 57.49, 43.19, 40.06, 39.97, 39.81, 39.72, 39.64, 39.56, 39.47, 39.30, 39.14, 38.96, 0.04	Matched m/z=322.1548. Predicted m/z=322.1550	93.22
2	Z4540473281	1H NMR (500 MHz, DMSO) δ 13.01 (s, 1H), 8.44 (t, J = 5.8 Hz, 1H), 8.14 (s, 2H), 7.81 – 7.75 (m, 2H), 7.75 – 7.62 (m, 2H), 7.02 – 6.90 (m, 2H), 6.53 – 6.44 (m, 2H), 3.92 (h, J = 6.8 Hz, 1H), 3.51 – 3.40 (m, 2H), 3.39 – 3.27 (m, 2H), 2.94 – 2.80 (m, 2H), 1.09 (d, J = 6.7 Hz, 3H)	13C NMR (126 MHz, DMSO) δ 166.56, 151.78, 136.19, 132.18, 131.92, 129.81, 128.46, 128.25, 128.23, 127.52, 127.48, 125.05, 124.61, 121.32, 120.88, 120.84, 119.42, 117.08, 116.80, 110.30, 106.91, 106.58, 101.56, 55.61, 50.53, 49.64, 49.14, 45.81, 45.50, 44.90, 42.26, 40.61, 40.52, 40.44, 40.35, 40.18, 40.11, 40.02, 39.85, 39.68, 39.52, 36.51, 28.14, 26.39, 22.99, 18.77, 14.71, 14.09, 0.58	Matched m/z=347.1859. Predicted m/z=347.1866	100.00
3	Z4540473138	1H NMR (400 MHz, DMSO-d6) δ 13.02 (s, 1H), 8.28 (s, 1H), 8.22 (d, J = 8.4 Hz, 1H), 8.03 – 7.97 (m, 1H), 7.81 – 7.73 (m, 2H), 7.72 – 7.63 (m, 2H), 7.34 (td, J = 8.6, 6.7 Hz, 1H), 7.15 (ddd, J = 10.4, 9.4, 2.6 Hz, 1H), 6.99 (tdd, J = 8.6, 2.7, 1.0 Hz, 1H), 4.35 – 4.19 (m, 1H), 2.84 (d, J = 7.0 Hz, 2H), 1.18 (d, J = 6.7 Hz, 3H)	13C NMR (101 MHz, DMSO) δ 165.69, 136.99, 136.14, 133.13, 133.07, 132.97, 132.24, 128.21, 126.55, 125.01, 122.74, 122.60, 120.89, 111.64, 111.42, 104.19, 103.94, 103.68, 45.78, 40.62, 40.42, 40.21, 40.00, 39.79, 39.58, 39.37, 34.82, 20.70	Matched m/z=342.1402. Predicted m/z=342.1412	100.00
4	Z4540472356	1H NMR (500 MHz, DMSO-d6) δ 13.02 (s, 1H), 8.29 (t, J = 5.9 Hz, 1H), 8.07 (s, 2H), 7.86 – 7.80 (m, 2H), 7.73 – 7.67 (m, 2H), 7.34 – 7.25 (m, 4H), 7.20 (ddt, J = 8.6, 5.9, 2.1 Hz, 1H), 3.22 (d, J = 5.9 Hz, 2H), 2.67 (s, 2H), 0.56 – 0.50 (m, 2H), 0.44 – 0.38 (m, 2H).	13C NMR (126 MHz, DMSO) δ 166.23, 139.66, 135.64, 131.77, 129.38, 127.95, 127.86, 125.88, 124.50, 120.38, 44.00, 40.06, 39.97, 39.89, 39.81, 39.72, 39.64, 39.56, 39.47, 39.30, 39.14, 38.97, 9.38, 0.03.	Matched m/z=332.1754. Predicted m/z=332.1757	91.21
5	Z4540473631	1H NMR (400 MHz, DMSO-d6) δ 13.02 (s, 1H), 8.52 (t, J = 4.7 Hz, 1H), 8.14 (s, 2H), 7.91 – 7.79 (m, 2H), 7.79 – 7.62 (m, 2H), 7.36 (td, J = 8.1, 6.0 Hz, 1H), 7.15 (d, J = 8.0 Hz, 1H), 7.02 (ddd, J = 9.5, 8.3, 1.1 Hz, 1H), 4.56 (dd, J = 4.7, 1.6 Hz, 2H), 2.48 (s, 3H)	13C NMR (101 MHz, DMSO) δ 166.23, 162.75, 160.30, 141.78, 136.24, 129.88, 129.79, 128.50, 124.99, 122.97, 122.81, 121.79, 120.89, 112.25, 112.01, 40.62, 40.42, 40.20, 40.00, 39.79, 39.58, 39.37, 35.53, 35.48, 15.77	Matched m/z=342.1070. Predicted m/z=342.1071	100.00
6	Z4540473602	1H NMR (400 MHz, DMSO-d6) δ 13.02 (s, 1H), 8.31 (dd, J = 15.9, 8.4 Hz, 2H), 8.00 (d, J = 8.3 Hz, 1H), 7.91 – 7.79 (m, 2H), 7.74 – 7.65 (m, 2H), 7.29 – 7.17 (m, 2H), 7.17 – 7.00 (m, 3H), 3.74 (tdd, J = 8.1, 6.5, 3.9 Hz, 1H), 1.97 (dt, J = 9.5, 5.2 Hz, 0H), 1.87 (dt, J = 9.3, 4.9 Hz, 1H), 1.37 – 1.24 (m, 4H), 1.07 – 0.90 (m, 1H), 0.94 – 0.84 (m, 1H)	13C NMR (101 MHz, DMSO) δ 165.69, 165.62, 143.60, 143.42, 137.00, 136.15, 132.38, 132.29, 128.70, 128.35, 128.31, 126.58, 126.08, 126.03, 125.76, 125.67, 125.03, 120.90, 48.92, 40.62, 40.42, 40.21, 40.00, 39.79, 39.59, 39.37, 29.93, 29.68, 21.60, 21.43, 20.84, 20.64, 14.72, 14.18	Matched m/z=332.1757. Predicted m/z=332.1757.	69.66
7	Z4540473457	1H NMR (400 MHz, DMSO-d6) δ 13.02 (s, 1H), 8.41 (t, J = 5.8 Hz, 1H), 8.29 (s, 1H), 8.01 (s, 1H), 7.87 – 7.79 (m, 2H), 7.74 – 7.66 (m, 2H), 7.50 (dd, J = 1.9, 0.8 Hz, 1H), 6.35 (dd, J = 3.1, 1.9 Hz, 1H), 6.18 (dd, J = 3.2, 0.8 Hz, 1H), 4.59 (t, J = 5.4 Hz, 1H), 3.37 (t, J = 5.3 Hz, 2H), 3.33 – 3.21 (m, 2H), 2.69 (dd, J = 15.3, 7.1 Hz, 1H), 2.60 (dd, J = 15.3, 6.6 Hz, 1H), 2.06 (ddd, J = 12.5, 7.2, 5.6 Hz, 1H)	13C NMR (101 MHz, DMSO) δ 166.76, 154.69, 141.79, 137.00, 136.22, 132.04, 128.24, 126.60, 125.09, 120.88, 110.80, 106.59, 61.87, 40.96, 40.78, 40.62, 40.42, 40.20, 40.00, 39.79, 39.58, 39.37, 27.84, 0.57	Matched m/z=348.1315. Predicted m/z=348.1319	100.00
8	Z4540472373	1H NMR (400 MHz, DMSO) δ 13.04 (s, 1H), 8.79 (t, J = 6.3 Hz, 1H), 8.29 (s, 1H), 8.02 (s, 1H), 7.87 – 7.75 (m, 1H), 7.75 – 7.63 (m, 1H), 7.61 (dd, J = 8.7, 5.4 Hz, 1H), 7.47 – 7.22 (m, 2H), 4.04 (td, J = 14.6, 6.2 Hz, 1H), 2.54 (s, 3H)	13C NMR (101 MHz, DMSO) δ 166.31, 163.06, 136.17, 131.69, 130.83, 128.01, 127.85, 124.64, 120.79, 120.32, 115.55, 44.63.	Matched m/z=368.0976. Predicted m/z=368.0981	100.00
9	Z4540473717	1H NMR (500 MHz, DMSO-d6) δ 13.03 (s, 1H), 8.90 (t, J = 5.8 Hz, 1H), 8.28 (s, 1H), 8.02 (s, 1H), 7.93 – 7.86 (m, 2H), 7.75 – 7.68 (m, 2H), 7.35 – 7.28 (m, 1H), 7.33 – 7.21 (m, 2H), 7.20 – 7.12 (m, 1H), 4.53 – 4.46 (m, 4H), 3.23 (t, J = 7.0 Hz, 2H), 2.48 (s, 1H), 2.32 (t, J = 8.1 Hz, 2H), 1.99 – 1.87 (m, 2H)	13C NMR (126 MHz, DMSO) δ 174.42, 166.29, 137.97, 136.41, 134.62, 131.79, 128.59, 128.38, 128.34, 127.81, 127.43, 125.17, 120.87, 46.71, 43.47, 40.61, 40.52, 40.44, 40.35, 40.30, 40.18, 40.11, 40.02, 39.85, 39.68, 39.51, 30.77, 17.88.	Matched m/z=375.1814. Predicted m/z=375.1816	97.20
10	Z4540473654	1H NMR (400 MHz, DMSO) δ 13.02 (s, 1H), 9.09 (s, 1H), 8.29 (s, 1H), 8.15 (d, J = 8.2 Hz, 1H), 8.00 (s, 1H), 7.90 – 7.81 (m, 2H), 7.74 – 7.65 (m, 2H), 7.03 – 6.94 (m, 2H), 6.70 – 6.61 (m, 2H), 4.00 (tt, J = 8.0, 6.0 Hz, 1H), 2.58 – 2.41 (m, 1H), 1.82 (dtd, J =16.9, 8.7, 6.5 Hz, 1H), 1.75 – 1.61 (m,1H), 1.16 (d, J = 6.6 Hz, 3H).	13C NMR (101 MHz, DMSO) δ 165.77, 155.71, 136.99, 136.06, 132.49, 132.43, 129.53, 128.31, 126.55, 125.01, 120.93, 115.48, 44.98, 40.62, 40.42, 40.20, 40.10, 40.00, 39.79, 39.58, 39.37, 38.58, 31.70, 21.28.	Matched m/z=336.1704. Predicted m/z=336.1707	100.00
11	Z4540473531	1H NMR (400 MHz, DMSO) δ 13.02 (s, 1H), 8.28 (s, 1H), 8.02 – 7.96 (m, 1H), 7.72 – 7.63 (m, 2H), 7.61 – 7.52 (m, 2H), 3.75 – 3.54 (m, 2H), 3.50 (s, 0H), 3.48 (s, 3H), 2.17 (q, J = 4.8 Hz, 2H), 1.72 (tdd, J = 11.8, 4.6, 2.8 Hz, 1H), 1.48 (ddq, J = 19.2, 11.5, 3.5 Hz, 2H), 1.41 – 1.22 (m, 3H), 1.22 – 1.04 (m, 1H), 0.72 (ddd, J = 12.2, 5.5, 2.3 Hz, 1H)	13C NMR (101 MHz, DMSO) δ 168.54, 136.95, 135.51, 132.17, 128.94, 126.52, 124.91, 120.90, 61.47, 47.10, 40.63, 40.42, 40.21, 40.00, 39.79, 39.58, 39.37, 38.92, 38.85, 38.34, 36.80, 34.28, 30.09, 28.95, 22.77.	Matched m/z=326.1860. Predicted m/z=326.1863	100.00
12	Z4540473508	1H NMR (400 MHz, DMSO-d6) δ 13.03 (s, 1H), 8.99 (t, J = 5.9 Hz, 1H), 8.30 (s, 1H), 8.02 (s, 1H), 7.94 – 7.86 (m, 2H), 7.75 – 7.68 (m, 2H), 7.39 (d, J = 1.0 Hz, 1H), 4.68 (s, 2H), 4.59 – 4.52 (m, 2H), 3.39 (s, 3H).	13C NMR (101 MHz, DMSO) δ 168.43, 166.28, 154.60, 137.03, 136.40, 131.75, 128.40, 126.63, 125.12, 120.87, 115.95, 71.06, 58.78, 40.62, 40.42, 40.21, 40.00, 39.79, 39.58, 39.37.	Matched m/z=329.1065. Predicted m/z=329.1067	100.00
13	Z4540472670	1H NMR (400 MHz, DMSO-d6) δ 12.90 (s, 1H), 10.27 (s, 1H), 8.11 (s, 1H), 7.94 (s, 1H), 7.81 – 7.66 (m, 4H), 7.64 – 7.56 (m, 2H), 7.53 (t, J = 7.9 Hz, 1H), 7.44 (ddd, J = 8.2, 2.4, 1.0 Hz, 1H), 3.84 (t, J = 6.5 Hz, 2H), 3.56 (t, J = 7.4 Hz, 2H), 2.51 – 2.39 (m, 2H).	13C NMR (101 MHz, DMSO) δ 165.37, 138.96, 137.37, 136.55, 129.66, 129.05, 125.71, 122.65, 121.38, 121.28, 117.58, 48.83, 47.07, 40.62, 40.42, 40.21, 40.00, 39.79, 39.58, 39.37, 19.01	Matched m/z=405.0989. Predicted m/z=405.0992	100.00
14	Z4540472173	1H NMR (400 MHz, DMSO-d6) δ 13.02 (s, 1H), 8.83 (t, J = 5.9 Hz, 1H), 8.29 (s, 1H), 8.01 (s, 1H), 7.92 – 7.83 (m, 2H), 7.75 – 7.66 (m, 2H), 7.61 (d, J = 2.1 Hz, 1H), 6.12 (d, J = 2.2 Hz, 1H), 4.42 (d, J = 5.8 Hz, 2H), 3.99 (t, J = 7.0 Hz, 2H), 1.76 (h, J = 7.3 Hz, 2H), 0.83 (t, J = 7.4 Hz, 3H)	13C NMR (101 MHz, DMSO) δ 166.13, 150.13, 137.01, 136.25, 131.97, 131.00, 128.34, 126.59, 125.09, 120.89, 103.98, 53.10, 40.62, 40.42, 40.21, 40.00, 39.79, 39.58, 39.37, 37.37, 23.72	Matched m/z=310.1661. Predicted m/z=310.1662	100.00

Supplementary Table 2-B: Chemical Characterization of ROCK1 Actives

Compound #	Enamine ID	1H NMR	13C NMR	HRMS (m/z)	Purity (HPLC area % at 254 nm)
15	Z3704235136	1H NMR (400 MHz, DMSO-d6) δ 13.02 (s, 1H), 8.83 (t, J = 5.9 Hz, 1H), 8.29 (s, 1H), 8.01 (s, 1H), 7.92 – 7.83 (m, 2H), 7.74 – 7.65 (m, 2H), 7.58 (d, J = 2.2 Hz, 1H), 6.12 (d, J = 2.1 Hz, 1H), 4.42 (d, J = 5.8 Hz, 2H), 3.84 (d, J = 7.2 Hz, 2H), 2.07 (dq, J = 13.7, 6.8 Hz, 1H), 0.83 (d, J = 6.7 Hz, 6H)	13C NMR (101 MHz, DMSO) δ 166.15, 150.17, 137.01, 136.25, 131.97, 131.44, 128.34, 126.60, 125.09, 120.89, 103.90, 58.80, 40.62, 40.41, 40.20, 40.00, 39.79, 39.58, 39.37, 37.37, 29.55, 20.17	Matched m/z=324.1815. Predicted m/z=324.1819	100.00
16	Z4540472138	1H NMR (500 MHz, DMSO-d6) δ 10.64 – 10.60 (m, 1H), 7.83 (d, J = 8.8 Hz, 1H), 7.78 (s, 1H), 7.46 (d, J = 0.7 Hz, 1H), 7.37 – 7.28 (m, 2H), 7.31 – 7.24 (m, 1H), 7.24 – 7.18 (m, 2H), 6.96 (dd, J = 7.2, 4.2 Hz, 1H), 6.78 (dd, J = 8.8, 2.3 Hz, 1H), 6.63 (t, J = 5.5 Hz, 1H), 6.55 (d, J = 2.2 Hz, 1H), 6.25 (d, J = 7.1 Hz, 1H), 5.28 (s, 2H), 4.17 (d, J = 5.4 Hz, 2H)	13C NMR (126 MHz, DMSO) δ 162.14, 152.08, 140.38, 139.02, 138.19, 129.54, 129.23, 128.94, 128.21, 128.03, 119.34, 116.31, 114.89, 104.90, 104.02, 55.21, 40.61, 40.52, 40.44, 40.36, 40.27, 40.18, 40.11, 40.02, 39.85, 39.68, 39.51, 37.51, 0.58	Matched m/z=331.1550. Predicted m/z=331.1553	100.00
17	Z4540473297	1H NMR (400 MHz, DMSO-d6) δ 9.20 (t, J = 5.9 Hz, 1H), 8.73 (s, 1H), 8.06 (dd, J = 1.5, 0.8 Hz, 1H), 7.97 (dd, J = 8.0, 1.4 Hz, 1H), 7.80 – 7.72 (m, 2H), 7.67 (q, J = 1.4 Hz, 1H), 4.53 – 4.46 (m, 2H), 4.43 (s, 2H)	13C NMR (101 MHz, DMSO) δ 169.64, 166.39, 141.27, 137.36, 130.60, 127.48, 127.41, 123.34, 123.18, 121.71, 45.45, 40.62, 40.41, 40.20, 39.99, 39.78, 39.57, 39.37, 38.67	Matched m/z=341.0565. Predicted m/z=341.0566	100.00
18	Z4540472556	1H NMR (400 MHz, DMSO) δ 9.20 (t, J = 6.0 Hz, 1H), 8.06 (t, J = 2.8 Hz, 1H), 8.09 – 7.67 (m, 3H), 7.67 – 7.34 (m, 4H), 7.03 (t, J = 55.9 Hz, 1H), 4.54 (d, J = 6.0 Hz, 2H), 3.40 (td, J = 6.6, 2.8 Hz, 2H), 2.96 (t, J = 6.6 Hz, 2H)	13C NMR (101 MHz, DMSO) δ 166.22, 164.31, 140.86, 139.88, 137.29, 134.58, 132.13, 130.33, 129.37, 127.59, 126.98, 125.95, 124.85, 124.80, 124.72, 124.65, 124.59, 115.44, 113.09, 42.94, 40.62, 40.42, 40.21, 40.00, 39.79, 39.58, 39.53, 39.37, 28.15.	Matched m/z=336.1704. Predicted m/z=336.1707	100.00
19	Z2505824737	1H NMR (400 MHz, DMSO) δ 7.76 (t, J = 2.6 Hz, 1H), 7.31 (tt, J = 8.4, 6.7 Hz, 1H), 7.20 – 6.97 (m, 4H), 6.65 (dd, J = 7.9, 1.4 Hz, 1H), 5.14 (t, J = 5.6 Hz, 1H), 3.32 (dd, J = 13.5, 2.7 Hz, 1H), 3.11 (q, J = 6.6 Hz, 2H), 2.73 (t, J = 7.6 Hz, 2H), 2.63 (t, J = 6.7 Hz, 2H), 1.94 – 1.78 (m, 2H)	13C NMR (101 MHz, DMSO) δ 165.71, 160.07, 145.42, 130.06, 128.83, 128.73, 128.63, 127.17, 124.33, 117.57, 115.38, 112.77, 111.98, 111.91, 111.80, 111.72, 43.30, 40.62, 40.42, 40.21, 40.00, 39.79, 39.58, 39.37, 39.01, 28.86, 22.56, 20.03.	Matched m/z=317.1453. Predicted m/z=317.1460	96.21
20	Z3496050817	1H NMR (500 MHz, DMSO-d6) δ 10.62 (s, 1H), 7.83 (d, J = 8.8 Hz, 1H), 7.65 (s, 1H), 6.96 (d, J = 7.2 Hz, 1H), 6.80 (dd, J = 8.8, 2.3 Hz, 1H), 6.56 (d, J = 2.3 Hz, 1H), 6.49 (t, J = 5.0 Hz, 1H), 6.27 (d, J = 7.1 Hz, 1H), 4.54 (p, J = 7.2 Hz, 1H), 4.06 (d, J = 5.0 Hz, 2H), 2.14 (s, 3H), 2.07 – 1.94 (m, 2H), 1.92 – 1.81 (m, 2H), 1.81 – 1.73 (m, 1H), 1.78 – 1.69 (m, 1H), 1.66 – 1.58 (m, 1H), 1.59 (s, 1H)	13C NMR (126 MHz, DMSO) δ 152.16, 145.87, 140.41, 129.21, 128.85, 128.15, 117.38, 116.18, 115.43, 114.83, 109.46, 104.91, 103.68, 62.08, 40.61, 40.52, 40.44, 40.36, 40.27, 40.18, 40.11, 40.02, 39.85, 39.68, 39.51, 37.27, 32.99, 24.16, 12.24, 0.58	Matched m/z=323.1860. Predicted m/z=323.1866	100.00
21	Z4540472882	1H NMR (400 MHz, DMSO-d6) δ 7.79 (d, J = 3.0 Hz, 1H), 7.52 (s, 4H), 7.15 (dd, J = 7.6, 1.2 Hz, 1H), 7.03 (t, J = 7.9 Hz, 1H), 6.54 (dd, J = 8.1, 1.2 Hz, 1H), 6.04 (t, J = 6.0 Hz, 1H), 4.44 (d, J = 5.9 Hz, 2H), 3.61 (s, 3H), 3.38 (td, J = 6.7, 2.8 Hz, 2H), 2.76 (t, J = 6.7 Hz, 2H)	13C NMR (101 MHz, DMSO) δ 165.62, 144.75, 130.15, 128.38, 127.64, 127.07, 126.85, 126.79, 126.73, 124.37, 115.64, 113.27, 52.13, 46.56, 40.62, 40.42, 40.20, 40.00, 39.79, 39.58, 39.37, 39.02, 22.75	Matched m/z=383.1368. Predicted m/z=383.1377	100.00
22	Z851078508	1H NMR (500 MHz, DMSO-d6) δ 11.70 (s, 1H), 8.30 – 8.20 (m, 2H), 7.57 (d, J = 2.8 Hz, 1H), 7.16 – 7.04 (m, 3H), 6.65 (d, J = 8.1 Hz, 2H), 6.62 – 6.55 (m, 1H), 6.21 (d, J = 3.2 Hz, 1H), 4.35 (s, 1H), 4.30 (s, 1H), 4.26 (s, 1H), 4.28 – 4.23 (m, 0H), 4.15 – 4.09 (m, 1H), 3.74 – 3.64 (m, 2H), 3.29 (s, 1H), 2.96 (s, 3H), 2.65 (q, J = 4.9, 4.2 Hz, 1H), 2.50 – 2.44 (m, 1H)	13C NMR (126 MHz, DMSO) δ 174.42, 166.29, 137.97, 136.41, 134.62, 131.79, 128.59, 128.38, 128.34, 127.81, 127.43, 125.17, 120.87, 46.71, 43.47, 40.61, 40.52, 40.44, 40.35, 40.30, 40.18, 40.11, 40.02, 39.85, 39.68, 39.51, 30.77, 17.88, 0.59	Matched m/z=347.1867. Predicted m/z=347.1866	100.00
23	Z1319102921	1H NMR (400 MHz, DMSO-d6) δ 11.69 (d, J = 4.4 Hz, 1H), 8.28 – 8.19 (m, 2H), 7.55 (dd, J = 9.4, 2.7 Hz, 1H), 7.10 (dt, J = 7.9, 4.4 Hz, 1H), 6.18 (dd, J = 10.2, 3.8 Hz, 1H), 6.02 – 5.87 (m, 2H), 4.17 (dq, J = 15.1, 2.8 Hz, 2H), 3.69 (dt, J = 14.1, 5.7 Hz, 2H), 2.81 (t, J = 7.5 Hz, 2H), 2.68 (ddd, J = 18.3, 8.9, 6.0 Hz, 2H), 2.57 (s, 1H), 2.20 (d, J = 2.8 Hz, 3H)	13C NMR (101 MHz, DMSO) δ 170.18, 153.55, 149.56, 143.26, 130.42, 130.05, 128.76, 128.70, 123.88, 123.75, 117.71, 117.26, 117.10, 116.23, 114.77, 106.65, 106.24, 44.66, 42.37, 42.12, 40.62, 40.41, 40.20, 39.99, 39.78, 39.57, 39.37, 38.38, 31.67, 31.21, 28.13, 27.36, 23.73, 23.64, 13.72	Matched m/z=336.1699. Predicted m/z=336.1707	98.47
24	Z3294657258	1H NMR (400 MHz, DMSO-d6) δ 11.69 (s, 1H), 8.23 (dq, J = 4.8, 3.0, 2.3 Hz, 2H), 7.55 (dd, J = 9.4, 2.6 Hz, 1H), 7.10 (dt, J = 8.2, 4.3 Hz, 1H), 6.17 (dt, J = 13.4, 3.4 Hz, 1H), 4.12 (h, J = 2.7 Hz, 2H), 3.73 – 3.56 (m, 4H), 3.06 – 2.83 (m, 1H), 2.57 – 2.45 (m, 2H), 2.32 – 2.11 (m, 3H), 1.99 – 1.73 (m, 4H)	13C NMR (101 MHz, DMSO) δ 172.11, 149.56, 143.26, 129.96, 128.76, 128.67, 123.88, 123.73, 117.79, 117.25, 117.17, 116.23, 114.78, 78.87, 67.09, 44.44, 42.27, 42.04, 40.62, 40.42, 40.20, 40.00, 39.79, 39.58, 39.37, 39.17, 38.70, 36.79, 28.35, 28.22, 27.84, 27.32, 25.22	Matched m/z=338.1855. Predicted m/z=338.1863	97.89
25	Z2633849139	1H NMR (400 MHz, DMSO) δ 12.85 (s, 1H), 10.75 (s, 1H), 8.62 – 8.55 (m, 1H), 7.97 (dd, J = 8.7, 1.7 Hz, 1H), 7.66 (d, J = 2.3 Hz, 1H), 7.49 (dd, J = 8.8, 0.8 Hz, 1H), 6.62 (d, J = 2.2 Hz, 1H), 4.06 (t, J = 7.2 Hz, 2H), 2.54 (s, 3H), 1.74 – 1.63 (m, 2H), 1.51 (dq, J = 13.3, 6.7 Hz, 1H), 0.91 (d, J = 6.6 Hz, 6H).	13C NMR (101 MHz, DMSO) δ 165.03, 147.77, 143.29, 142.37, 130.53, 126.21, 125.92, 122.27, 121.30, 110.07, 97.50, 49.85, 40.62, 40.42, 40.21, 40.00, 39.79, 39.58, 39.37, 39.16, 25.38, 22.68, 12.13.	Matched m/z=312.1815. Predicted m/z=312.1819.	96.55
26	Z2636445892	1H NMR (400 MHz, DMSO-d6) δ 12.81 (s, 1H), 8.13 (d, J = 8.2 Hz, 1H), 7.98 – 7.92 (m, 1H), 7.75 – 7.67 (m, 2H), 7.61 (dd, J = 8.8, 1.6 Hz, 1H), 7.42 (dd, J = 8.7, 0.8 Hz, 1H), 7.34 – 7.26 (m, 2H), 4.47 – 4.35 (m, 1H), 3.72 (dd, J = 14.6, 8.3 Hz, 1H), 3.48 (dd, J = 14.5, 4.3 Hz, 1H), 2.52 (s, 3H), 2.16 (s, 3H), 1.22 (d, J = 6.7 Hz, 3H)	13C NMR (101 MHz, DMSO) δ 165.88, 144.52, 142.85, 142.21, 137.14, 130.12, 128.16, 126.25, 125.73, 121.98, 120.16, 109.74, 59.98, 41.60, 40.62, 40.42, 40.21, 40.00, 39.79, 39.58, 39.37, 21.50, 21.28, 12.20	Matched m/z=372.1375. Predicted m/z=372.1376	100.00
27	Z2632659156	1H NMR (400 MHz, DMSO) δ 12.93 (s, 1H), 10.13 (s, 1H), 8.43 (t, J = 1.2 Hz, 1H), 7.91 (dd, J = 8.7, 1.7 Hz, 1H), 7.58 – 7.51 (m, 1H), 5.90 (s, 1H), 3.92 (q, J = 7.2 Hz, 2H), 2.56 (s, 3H), 1.84 (tt, J = 8.4, 5.0 Hz, 1H), 1.28 (t, J = 7.2 Hz, 3H), 0.89 – 0.78 (m, 2H), 0.70 – 0.58 (m, 2H)	13C NMR (101 MHz, DMSO) δ 166.56, 152.56, 143.31, 142.55, 136.28, 126.09, 125.31, 122.23, 121.56, 110.34, 97.60, 42.90, 40.62, 40.42, 40.20, 40.00, 39.79, 39.58, 39.37, 15.42, 12.20, 9.94, 8.09.	Matched m/z=310.1663. Predicted m/z=310.1662	100.00

Supplementary Note 1: Selection of Protein Structure for Docking

Eighteen ROCK1 protein structures were exported from the protein kinase database within MOE (Chemical Computing Group), which contains curated, pre-aligned kinase structures. The protein databank codes for the structures were: 2ESM, 2ETK, 2ETR, 2V55, 3D9V, 3NCZ, 3NDM, 3TV7, 3TWJ, 3V8S, 4L6Q, 4W7P, 4YVC, 4YVE, 5BML, 5HVU, 5KKS, and 5KKT. Those were further examined in detail using SeeSAR (version 9.2, BioSolveIT GmbH, Sankt Augustin, Germany, 2020, www.biosolveit.de/SeeSAR) with the goal to find one structure suitable to re-dock all 18 co-crystalized reference compounds into it with reasonable accuracy and associated affinity estimates. This kind of validation is crucial as throughout the process, the number of compounds to be docked reaches several millions. So, for practical reasons they can only be docked into one structure; but also, for statistical reasons, the best possible structure for scoring has to be chosen, as selecting just 100 from millions requires a close to perfect enrichment ratio of actives over inactives chosen.

To find the most suitable structure, the binding modes of the co-crystalized ligands in all 18 crystal structures were visually inspected. All of them share an H-bond interaction with the backbone NH of Met323. Several ligands interact additionally with the neighbouring backbone carbonyl of Glu321, forming a solid hinge binding motif. Further H-bonds are made with the gatekeeper or DFG-loop amino acids, further away from the hinge, which makes sense due to the drug-like size of the crystalized molecules. As in the first step of the Chemical Space Docking only fragment-sized molecules will be docked, placement constraints need to focus on one region only, as otherwise, the fragments may not be able to span the distance between them. Therefore, multiple alternative pharmacophoric constraints were defined, but all only in the hinge region:

- Essential H-Bond-Acceptor opposite of the backbone nitrogen of Met323
- H-Bond-Donor opposite of the backbone carbonyl of Glu321 or H-Bond-Acceptor opposite of the backbone nitrogen of Met323, but at least one of them needed to be fulfilled
- No constraints.

To find a good docking setup, the 18 co-crystalized ligands were docked into all 18 crystal structures applying the three described pharmacophore constraints. Binding sites on the crystal structures were determined using the binding site mode in SeeSAR (version 9.2) by copying all 18 ligands into that mode and picking the amino acids within 6.5 Å distance around the union of all ligand atoms.

Best results were obtained with the second setup in the crystal structures 2ETR, 2V55, 4YVC, 5KTT. The superposition of those was visually inspected and 2V55 was dismissed as it shows big differences to the other structures and hence did not seem to be a good consensus structure. As the remaining three are very similar the electron density was checked using EDIA.[1] Results are shown in Supplementary Figure 1. EDIA represents the electron density by different colors, areas which show high electron density are depicted in blue, whilst areas with low density are highlighted in red. The three remaining crystal structures are all well resolved in the hinge area, but overall 2ETR shows the

biggest blue areas, hence is the most trustworthy structure. Due to the good docking results in structure 2ETR (res. 2.6 Å) under application of pharmacophore constraint 2, this setup is used for the chemical space docking

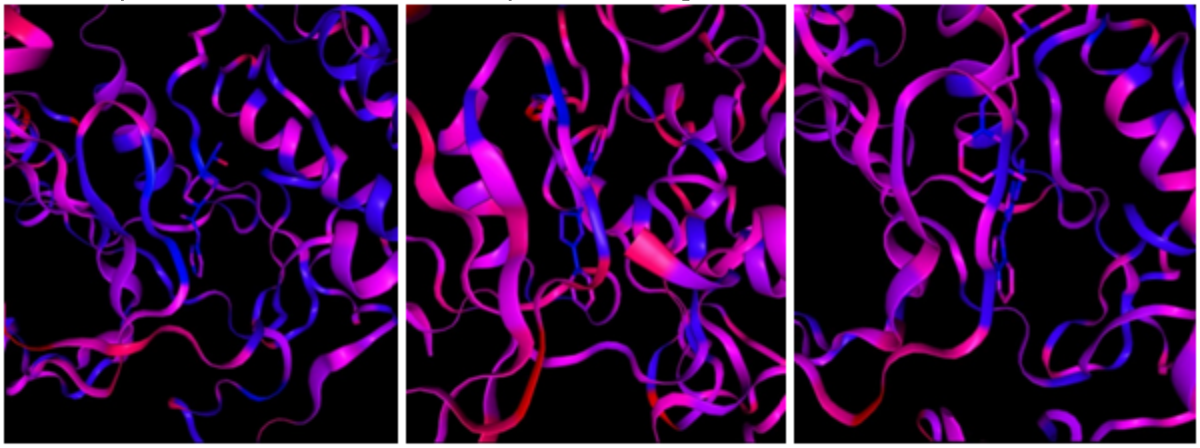

Supplementary Figure 1: Electron densities of ROCK-1 crystal structures: PDB ID 2ETR (left), 4YVC (middle), 5KKT (right). Areas with high electron density are depicted in blue, areas with a low density in red.

Supplementary Note 2: Structural Biology Details

Supplementary Table 3: Data Collection and Refinement Statistics

	ROCK1-Cmp 1 (7S25)	ROCK1-Cmp 22 (7S26)
**Data collection**		
Space group	$P\ 2_1$	$P\ 2_1$
Cell dimensions		
a, *b*, *c* (Å)	57.03, 100.96, 176.22	57.14, 101.60, 182.66
α, β, γ (°)	90, 90.84, 90	90, 91.98, 90
Resolution (Å)	2.34 (2.38-2.34)*	2.74 (2.79-2.74)
R_{sym} or R_{merge}	0.039 (1.076)	0.053 (0.952)
$I / \sigma I$	16.2 (1.4)	14.4 (1.3)
Completeness (%)	98.3 (99.6)	99.9 (99.9)
Redundancy	4.3 (4.3)	4.3 (4.0)
**Refinement**		
Resolution (Å)	2.34	2.74
No. reflections	82006/1238	54015/733
R_{work} / R_{free}	21.7/25.6	24.3/27.6
No. atoms		
Protein	11226	11231
Ligand	72	78
Water	143	37
B-factors	(Ask for input)	
Protein	45.3	54.8
Ligand/ion	75.1	104.4
Water	71.6	101.0
R.m.s. deviations		
Bond lengths (Å)	0.017	0.014
Bond angles (°)	1.95	1.74

*Values in parentheses are for highest-resolution shell.

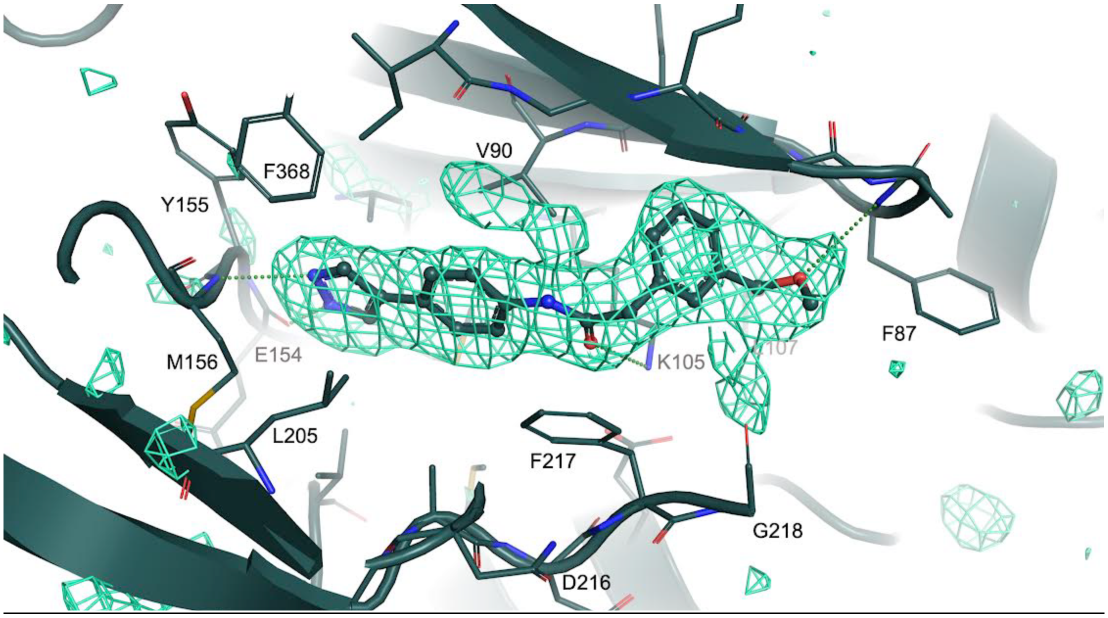

Supplementary Figure 2: Polder omit map for **1** calculated with Phenix by omitting all copies of the ligand and local bulk solvent masks. The mFo-DFc omit difference density is shown at 3 sigma contour level.

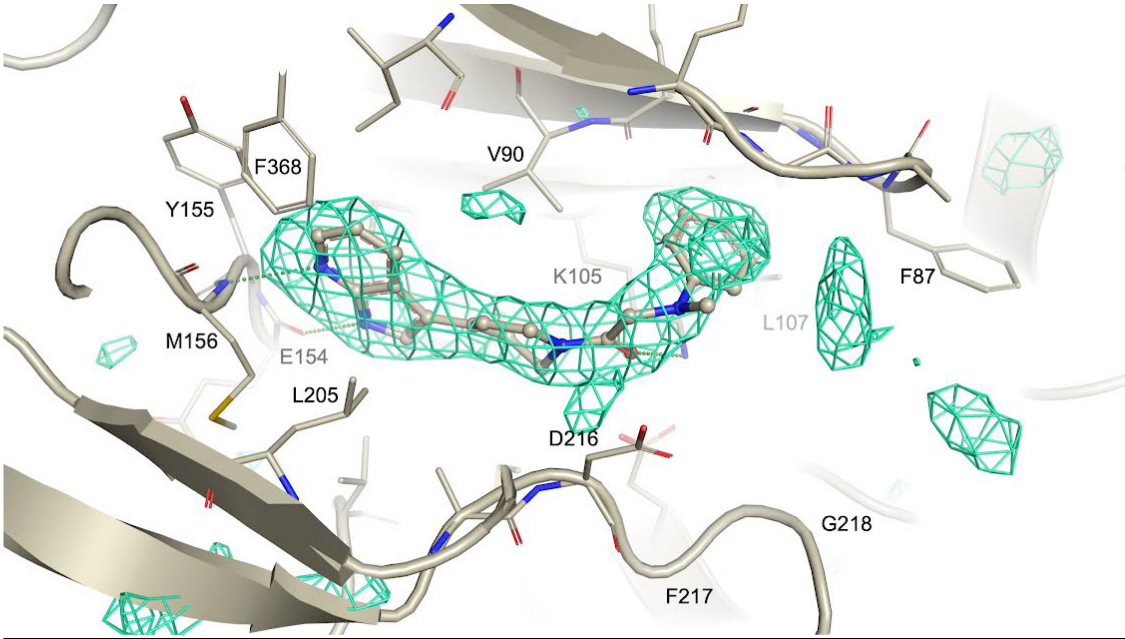

Supplementary Figure 3: Polder omit map for **22** calculated with Phenix by omitting all copies of the ligand and local bulk solvent masks. The mFo-DFc omit difference density is shown at 3 sigma contour level.

Supplementary Note 3: Comparison with an alternative ligand-based virtual screening approach

When structure-based virtual screening is considered too expensive, multi-step filter cascades are often times considered the only viable alternative. Fast methods are then used to trim down the massive amounts of data and slower methods are used towards the end of the funnel. 2D fingerprint similarity is an extremely fast approach for such filtering. In order to test the performance of this methodology, we collected a set of highly active ROCK1 inhibitors from the Chembl database. We first assessed the similarity of these compounds compared to our hit-set, using ECFP4 fingerprints and the SpaceLight program.[2] The respective highest similarity values of a hit compared to any of the Chembl actives cover a significant range from about 0.3 to 0.6. We then used each one of the Chembl actives in turn to retrieve all compound from the version of REAL Space used here throughout, with a 2D similarity >0.25. These searches resulted in a total of 6 mio compounds. Supplementary Figure 4 shows the distribution of 2D similarities in this set, as well as the similarity to each one of the hits presented herein.

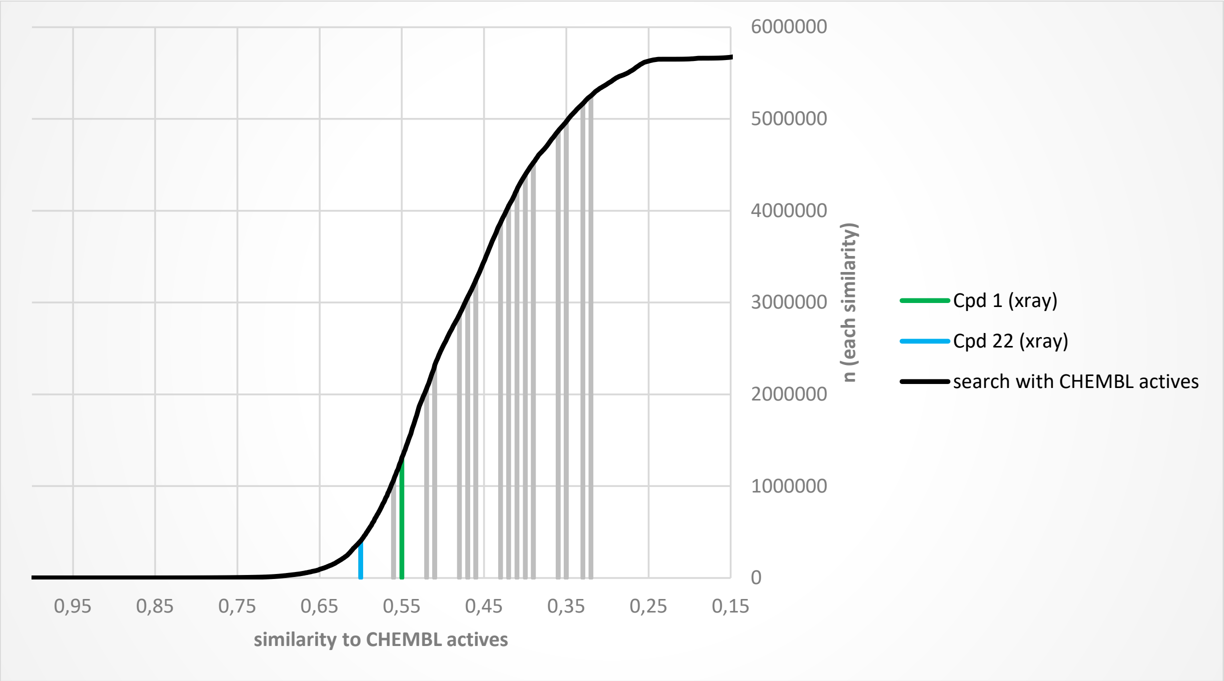

Supplementary Figure 4: distribution of similarities in REAL Space compared to Chembl ROCK1 actives together with the respective highest similarity compared to the hit molecules presented here. Source data are provided as a Source Data file.

Several observations are noteworthy. (1) the number of molecules retrieved this way is on the same order of magnitude as the number of molecules docked in the Chemical Space Docking process, so the computational effort is not significantly lower and the approach is certainly not trivial. (2) the range of similarities is quite wide and the distribution of similarities to the novel hits is quite even. So there is no particular bias

towards higher similarities in the hit-set, which supports the hypothesis that docking helps avoid such bias. (3) the number and range of similarities in the set of Chembl ROCK1 actives is quite large as well. So the alternative approach might be viable here but most certainly not on targets without such a rich body of data, as is available for ROCK1.

Supplementary Note 4: Comparison to Fully Enumerated Docking

In the Chemical Space Docking approach, the search space is significantly pruned by selecting building blocks that dock well to the binding site. The chemical subspace associated with each building block is fully enumerated through its associated reaction and all complementary building block partners. But how do the results compare with those obtained by brute-force, fully enumerated docking?

A docking campaign that enumerates the full library would be extremely time-consuming and expensive, but we conducted two types of experiments to compare our results to exhaustive docking. In the first approach, we randomly sampled 1% of the compounds from all sub-libraries, giving a sample size that is 1% of the total enumerated set and evenly distributed across all reactions in the chemical space. The total number of docked molecules in Chemical Space Docking and the random sample of 1% of the entire space is roughly the same, about 8 million.

We compared the distribution of docking scores from the random sample to those obtained from Chemical Space Docking (as described in the manuscript). Supplementary Figure 5 shows the comparison of the distribution of docking scores of both samples. The vast majority of the compounds sampled from the full library are poor dockers, while Chemical Space Docking significantly enriches for good dockers. This demonstrates that Chemical Space Docking is much more efficient at finding compounds that have high docking scores.

In the second approach, we selected two chemical subspaces (reactions and associated building blocks) from which experimentally validated hits were obtained and whose size reasonably allowed fully enumerated docking: subspaces s38 (~3.8 million compounds) and s270302 (~838 thousand compounds). We enumerated and docked all compounds in these subspaces and compared the distribution of docking scores to those selected by Chemical Space Docking.

Supplementary Figures 6 and 7 show the comparisons of the Chemical Space Docking results to the full enumeration of the compounds in subspaces s38 and s270302. For subspace s38 (Supplementary Figure 6) the chemical space docking approach does a very good job retrieving the best dockers from the full-enumeration docking, and its distribution of scores almost matches a very sparsely populated tail of the full distribution. A striking amount of unproductive effort is avoided by limiting the very poor dockers (score <1) to only a few hundred.

Chemical subspace s270302 (Supplementary Figure 7) looks a little different. Again we see a significant waste of effort in the > 95% of the fully enumerated library that fails to

achieve a reasonable docking score. A comparison of the distributions of the high scoring compounds shows that, for this subspace, Chemical Space Docking does indeed miss some of the compounds that were identified by docking the fully enumerated library. This results not from a failure to identify an initial building block as a good docking component, but rather from the filtering using the six exclusion criteria, as detailed in the main manuscript. This criteria clearly excluded initial building blocks that led to complete products that docked well, but whose instantiation was never realized because the building blocks were filtered out beforehand.

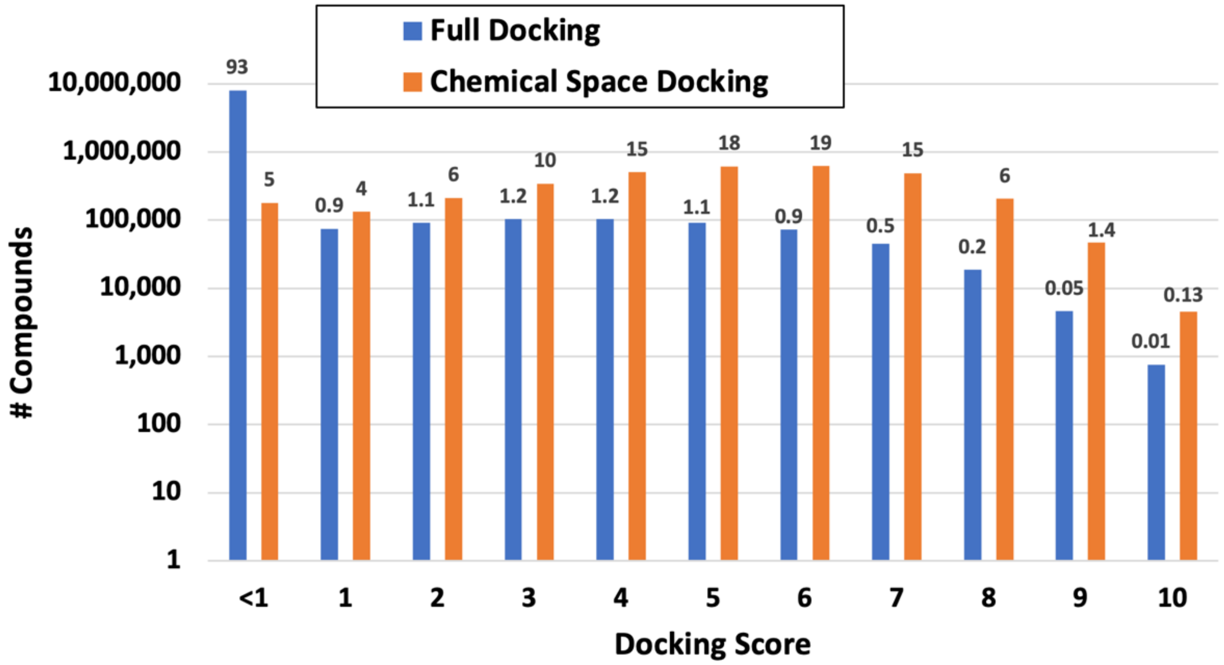

Supplementary Figure 5: Comparison of the distribution of docking scores from chemical space docking with that obtained from a random sample of 1% of the full chemical space. Docking scores are binned in 1 unit increments of estimated pIC50 values and reported as a histogram. Bars are labeled by the percentage of the compounds in the sampled chemical space. Source data are provided as a Source Data file.

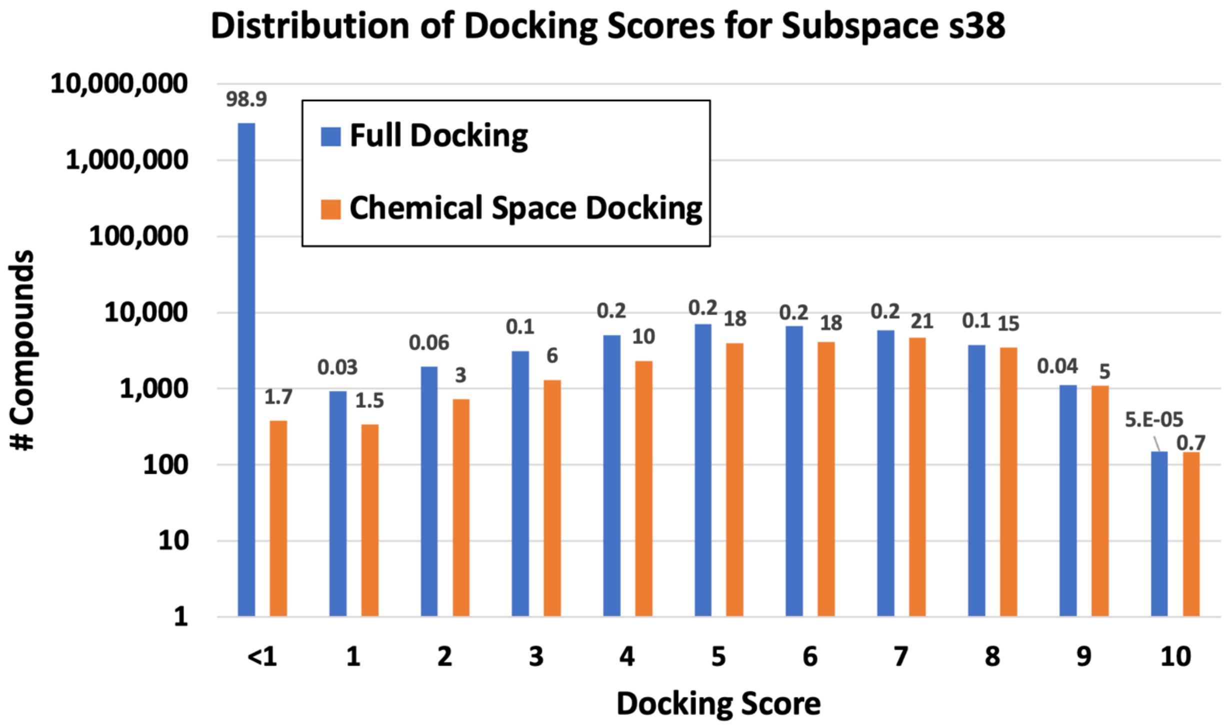

Supplementary Figure 6: Comparison of the docking score distributions for chemical subspace s38. Docking scores are binned in 1 unit increments of estimated pIC50 values and reported as a histogram. Bars are labeled by the percentage of the compounds in the sampled chemical space. Source data are provided as a Source Data file.

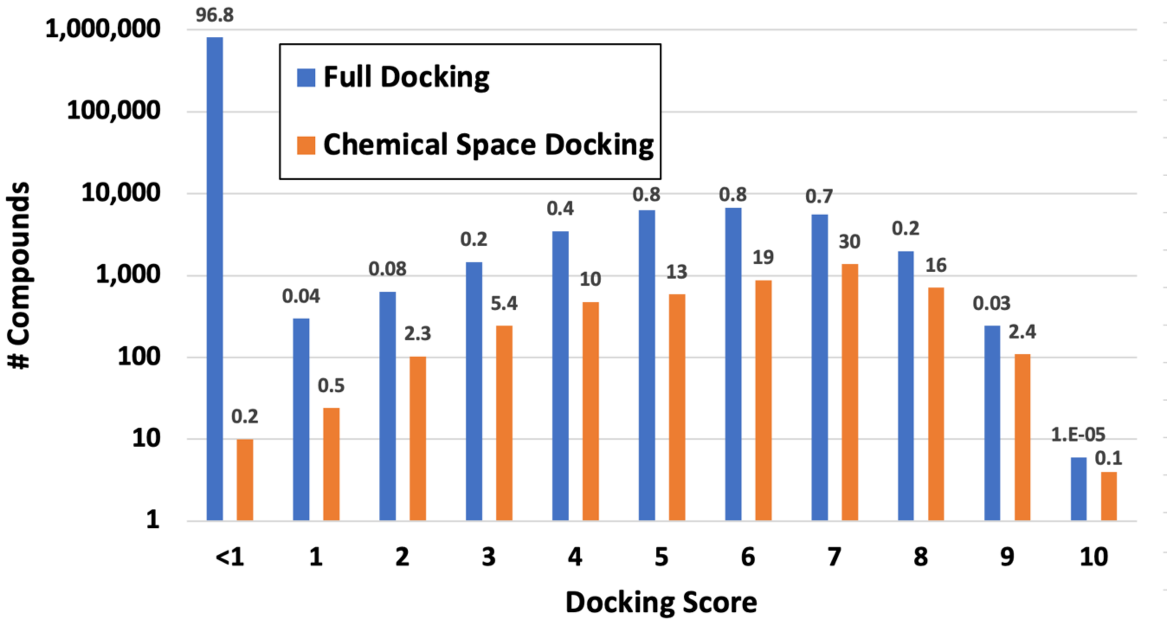

Supplementary Figure 7: Comparison of the docking score distributions for chemical subspace s270302. Docking scores are binned in 1 unit increments of estimated pIC50 values and reported as a histogram. Bars are labeled by the percentage of the

compounds in the sampled chemical space. Source data are provided as a Source Data file.

Supplementary References

(1) Meyder, A., Nittinger, E., Lange, G., Klein, R., and Rarey, M. (2017) Estimating Electron Density Support for Individual Atoms and Molecular Fragments in X-ray Structures. *J Chem Inf Model 57*, 2437–2447.

(2) Bellmann, L., Penner, P., and Rarey, M. (2021) Topological Similarity Search in Large Combinatorial Fragment Spaces. *J Chem Inf Model 61*, 238–251.
