# Supplementary material for: Chemical space docking enables large-scale structure-based virtual screening to discover ROCK1 kinase inhibitors
Source: Nat Commun. 2022 Oct 28;13:6447. doi: 10.1038/s41467-022-33981-8 (PMC9616902; doi:10.1038/s41467-022-33981-8)
Supplement: Supplementary file 3 — Reporting Summary [file 41467_2022_33981_MOESM3_ESM.pdf]

nature portfolio

Corresponding author(s): Paul Beroza

Last updated by author(s): Aug 24, 2022

Reporting Summary

Nature Portfolio wishes to improve the reproducibility of the work that we publish. This form provides structure for consistency and transparency in reporting. For further information on Nature Portfolio policies, see our Editorial Policies and the Editorial Policy Checklist.

Statistics

For all statistical analyses, confirm that the following items are present in the figure legend, table legend, main text, or Methods section.

n/a	Confirmed	
☐	☒	The exact sample size (*n*) for each experimental group/condition, given as a discrete number and unit of measurement
☒	☐	A statement on whether measurements were taken from distinct samples or whether the same sample was measured repeatedly
☒	☐	The statistical test(s) used AND whether they are one- or two-sided<br>*Only common tests should be described solely by name; describe more complex techniques in the Methods section.*
☒	☐	A description of all covariates tested
☒	☐	A description of any assumptions or corrections, such as tests of normality and adjustment for multiple comparisons
☐	☒	A full description of the statistical parameters including central tendency (e.g. means) or other basic estimates (e.g. regression coefficient) AND variation (e.g. standard deviation) or associated estimates of uncertainty (e.g. confidence intervals)
☒	☐	For null hypothesis testing, the test statistic (e.g. *F*, *t*, *r*) with confidence intervals, effect sizes, degrees of freedom and *P* value noted<br>*Give P values as exact values whenever suitable.*
☒	☐	For Bayesian analysis, information on the choice of priors and Markov chain Monte Carlo settings
☒	☐	For hierarchical and complex designs, identification of the appropriate level for tests and full reporting of outcomes
☒	☐	Estimates of effect sizes (e.g. Cohen's *d*, Pearson's *r*), indicating how they were calculated

Our web collection on statistics for biologists contains articles on many of the points above.

Software and code

Policy information about availability of computer code

Data collection	FlexX version 4.3 (BioSolveIT GmbH, Sankt Augustin, Germany, www.biosolveit.de)<br>OEDocking version 1.4.1
Data analysis	SeeSAR version 10.0 (BioSolveIT GmbH, Sankt Augustin, Germany, www.biosolveit.de)<br>Vortex version 2018.03.71496.53-s (Dotmatics, Inc. Bishops Stortford, United Kingdom, www.dotmatics.com)<br>MOE version 2019.0104 (Chemical Computing Group, Montreal, Canada, www.chemcomp.com)<br>Chemalot (downloaded May 2020) https://github.com/chemalot/chemalot<br>GeneData Screener v17 (Genedata AG, Basel, Switzerland, www.genedata.com)<br>MOLREP 11.0 (https://www.ccp4.ac.uk/)<br>COOT 0.9.6 (https://www2.mrc-lmb.cam.ac.uk/personal/pemsley/coot/)<br>REFMAC 5.8.0258 ((https://www2.mrc-lmb.cam.ac.uk/personal/pemsley/coot/)

For manuscripts utilizing custom algorithms or software that are central to the research but not yet described in published literature, software must be made available to editors and reviewers. We strongly encourage code deposition in a community repository (e.g. GitHub). See the Nature Portfolio guidelines for submitting code & software for further information.

Data

Policy information about availability of data

All manuscripts must include a data availability statement. This statement should provide the following information, where applicable:

- Accession codes, unique identifiers, or web links for publicly available datasets
- A description of any restrictions on data availability
- For clinical datasets or third party data, please ensure that the statement adheres to our policy

Source data are provided with this paper. Chemical structures and their analytical and biological characterization are presented in the manuscript and its Supplemental Information. Crystal structure coordinates and structure factors for compounds 1 and 22 are deposited in the PDB under accession codes 7S25 [http://doi.org/10.2210/pdb7S25/pdb] and 7S26 [http://doi.org/10.2210/pdb7S26/pdb].

Human research participants

Policy information about studies involving human research participants and Sex and Gender in Research.

Reporting on sex and gender	n/a
Population characteristics	n/a
Recruitment	n/a
Ethics oversight	n/a

Note that full information on the approval of the study protocol must also be provided in the manuscript.

Field-specific reporting

Please select the one below that is the best fit for your research. If you are not sure, read the appropriate sections before making your selection.

☒ Life sciences ☐ Behavioural & social sciences ☐ Ecological, evolutionary & environmental sciences

For a reference copy of the document with all sections, see nature.com/documents/nr-reporting-summary-flat.pdf

Life sciences study design

All studies must disclose on these points even when the disclosure is negative.

Sample size	The sample size for Full Docking (Supplementary Figure 5) was 1% of the fully enumerated Enamine REAL compounds, which was approximately equal to the initial population of products chosen by Chemical Space Docking (before applying filters for final selections). The sample sizes for Supplementary Figures 6 and 7 were determined by the number of reagents associated with reactions s38 and s270302 in the Enamine compound space.
Data exclusions	No data were excluded from analysis
Replication	Table 1: The data are the mean ± standard deviaion from three independent assays. There were no failures to replicate assay results.
Randomization	The 1% of the compound selected for full docking in Supplemental Figure 5 was chosen randomly.
Blinding	No chemical structures, only compound IDs, were provided with the samples for assay.

Reporting for specific materials, systems and methods

We require information from authors about some types of materials, experimental systems and methods used in many studies. Here, indicate whether each material, system or method listed is relevant to your study. If you are not sure if a list item applies to your research, read the appropriate section before selecting a response.

Materials & experimental systems

n/a	Involved in the study
☐	☒ Antibodies
☒	☐ Eukaryotic cell lines
☒	☐ Palaeontology and archaeology
☒	☐ Animals and other organisms
☒	☐ Clinical data
☒	☐ Dual use research of concern

Methods

n/a	Involved in the study
☒	☐ ChIP-seq
☒	☐ Flow cytometry
☒	☐ MRI-based neuroimaging

Antibodies

Antibodies used	An STK antibody is contained in commerically available HTRF assay kit (Cisbio-62ST2PEC) that was used to measure the biological activity of the synthesized compounds.
Validation	*Describe the validation of each primary antibody for the species and application, noting any validation statements on the manufacturer's website, relevant citations, antibody profiles in online databases, or data provided in the manuscript.*
